# Histone Deacetylases Enhance Ca^2+^-Activated K^+^ Channel K_Ca_3.1 Expression in Murine Inflammatory CD4^+^ T Cells

**DOI:** 10.3390/ijms19102942

**Published:** 2018-09-27

**Authors:** Miki Matsui, Kyoko Terasawa, Junko Kajikuri, Hiroaki Kito, Kyoko Endo, Pattaporn Jaikhan, Takayoshi Suzuki, Susumu Ohya

**Affiliations:** 1 Department of Pharmacology, Division of Pathological Sciences, Kyoto Pharmaceutical University, Kyoto 607-8414, Japan; kd15009@poppy.kyoto-phu.ac.jp (M.M.); kyoko.terasawa@outlook.jp (K.T.); kd16001@poppy.kyoto-phu.ac.jp (K.E.); 2 Department of Pharmacology, Graduate School of Medical Sciences, Nagoya City University, Nagoya 467-8601, Japan; kajikuri@med.nagoya-cu.ac.jp (J.K.); kito@med.nagoya-cu.ac.jp (H.K.); 3 Graduate School of Medical Science, Kyoto Prefectural University of Medicine, Kyoto 403-8334, Japan; pjaikhan@koto.kpu-m.ac.jp (P.J.); suzukit@koto.kpu-m.ac.jp (T.S.)

**Keywords:** Ca^2+^-activated K^+^ channel, K_Ca_3.1, histone deacetylase, inflammatory CD4^+^ T cell, inflammatory bowel disease

## Abstract

The up-regulated expression of the Ca^2+^-activated K^+^ channel K_Ca_3.1 in inflammatory CD4^+^ T cells has been implicated in the pathogenesis of inflammatory bowel disease (IBD) through the enhanced production of inflammatory cytokines, such as interferon-γ (IFN-γ). However, the underlying mechanisms have not yet been elucidated. The objective of the present study is to clarify the involvement of histone deacetylases (HDACs) in the up-regulation of K_Ca_3.1 in the CD4^+^ T cells of IBD model mice. The expression levels of K_Ca_3.1 and its regulators, such as function-modifying molecules and transcription factors, were quantitated using a real-time polymerase chain reaction (PCR) assay, Western blotting, and depolarization responses, which were induced by the selective K_Ca_3.1 blocker TRAM-34 (1 μM) and were measured using a voltage-sensitive fluorescent dye imaging system. The treatment with 1 μM vorinostat, a pan-HDAC inhibitor, for 24 h repressed the transcriptional expression of K_Ca_3.1 in the splenic CD4^+^ T cells of IBD model mice. Accordingly, TRAM-34-induced depolarization responses were significantly reduced. HDAC2 and HDAC3 were significantly up-regulated in the CD4^+^ T cells of IBD model mice. The down-regulated expression of K_Ca_3.1 was observed following treatments with the selective inhibitors of HDAC2 and HDAC3. The K_Ca_3.1 K^+^ channel regulates inflammatory cytokine production in CD4^+^ T cells, mediating epigenetic modifications by HDAC2 and HDAC3.

## 1. Introduction

The intermediate-conductance Ca^2+^-activated K^+^ channel K_Ca_3.1 controls Ca^2+^-dependent signaling pathways and plays crucial roles in proliferation, migration, apoptosis, and cytokine production in T cells [1,2,3,4]. Previous studies demonstrated the involvement of K_Ca_3.1 in the pathogenesis of inflammatory bowel disease (IBD) [5,6,7,8]. Concomitant with these studies, a recent clinical study showed that the expression levels of K_Ca_3.1 transcripts were higher in IBD patients than in controls [9]. Therefore, K_Ca_3.1 is a potent therapeutic target in IBD, in addition to T-cell-mediated autoimmune diseases, such as multiple sclerosis and rheumatoid arthritis. The K_Ca_3.1 auxiliary subunits that positively or negatively control its activity as well as T-cell function have been identified: phosphoinositide-3-kinase, class 2, β polypeptide (PI3K-C2B), nucleoside diphosphate kinase-B (NDPK-B), phosphohistidine phosphatase 1 (PHPT1), myotubularin-related protein 6 (MTMR6), tripartite motif containing-27 (TRIM-27), and phosphoglycerate mutase family member 5 (PGAM5) [8,10,11,12,13,14].

To date, several transcriptional regulators of K_Ca_3.1 have been identified in various cell types: activator protein-1 (AP-1) [15,16,17,18], repressor element silencing transcription factor/neuron-restrictive silencer factor (REST/NRSF) [19,20], and histone deacetylases [21]. AP-1 is a transcriptional factor that consists of a homodimer or heterodimer of Fos (c-Fos, FosB, Fra-1, Fra-2)/Jun (c-Jun, JunB, JunD). AP-1 is responsible for mitogen-induced K_Ca_3.1 up-regulation in T cells [15], IL-4 receptor signal-induced K_Ca_3.1 up-regulation in microglia [18], and angiotensin II-induced K_Ca_3.1 up-regulation in cardiac fibroblasts [16]. REST is a transcriptional repressor, and the up-regulation of K_Ca_3.1 mediates the down-regulation of REST which, in turn, promotes hyperplasia in vascular smooth muscle cells [19] and prostatic stromal cells [20]. HDACs are epigenetic regulators that consist of 11 members and are inhibited by the pan-HDAC inhibitor (HDACi), vorinostat. We previously reported the involvement of HDACs (HDAC2 and HDAC3) in K_Ca_3.1 transcription in human breast cancer cells [21].

The application of HDACis to the treatment of various types of disorders, including immunological and inflammatory disorders, is currently being investigated. The administration of broad-acting HDACis, such as vorinostat, meliorated intestinal inflammation by down-regulating inflammatory cytokines, including IFN-γ, in the CD4^+^ T cells of IBD model mice and suppressing their mobilization and accumulation [22,23]. Significant elevations in histone acetylation have been observed in the inflamed mucosa of IBD models and inflamed biopsies from patients with Crohn’s disease (CD) [24]. HDAC2, 3, 6, 9, and 10 have been proposed as the HDAC isoforms that are involved in chronic intestinal inflammation [25]. HDACs also play an important role in differentiation into anti-inflammatory cytokine-producing regulatory T (T_reg_) cells, and HDACis ameliorate autoimmune colitis through the induction of these cells [26,27,28]. Therefore, HDACis represent a potential therapeutic strategy in patients with IBD.

Our previous findings suggested that the up-regulation of K_Ca_3.1 in inflammatory CD4^+^ T cells is involved in the pathogenesis of IBD by promoting the expression and production of IFN-γ. In the present study, the mechanisms underlying the up-regulation of K_Ca_3.1 in the CD4^+^ T cells of IBD model mice were investigated.

## 2. Results

### 2.1. Up-Regulation of HDAC2 and 3 in CD4^+^ T Cells of Dextran Sulfate Sodium (DSS)-Induced Inflammatory Bowel Disease (IBD) Model Mice

**Figure 1:**
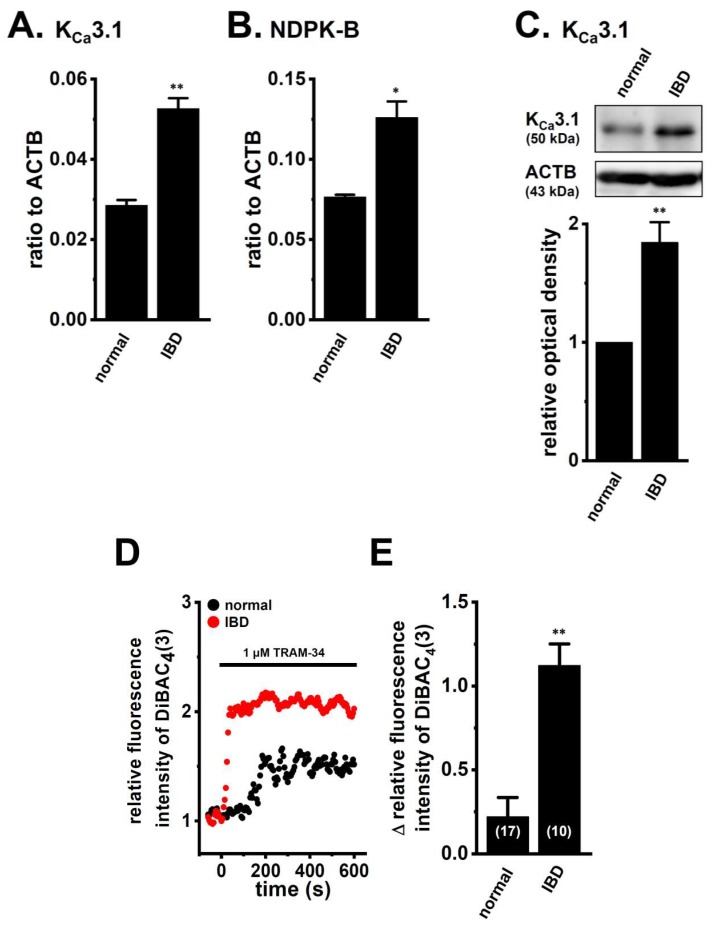
Increase in the expression and activity of K_Ca_3.1 in splenic CD4^+^ T cells of IBD model mice. (**A**,**B**) Real-time PCR assay for K_Ca_3.1 (**A**) and NDPK-B (**B**) in the splenic CD4^+^ T cells of “normal” and “IBD” model mice (*n* = 4 for each). Expression levels were expressed as a ratio to ACTB. (**C**) Expression of K_Ca_3.1 proteins (approximately 50 kDa) in the splenic CD4^+^ T cells of “normal” and “IBD” model mice. Protein lysates of the examined cells were probed by immunoblotting with anti-K_Ca_3.1 (upper panel) and anti-ACTB (lower panel) antibodies on the same filter. Summarized results were obtained as the optical density of K_Ca_3.1 and ACTB band signals. After compensation for the optical density of the K_Ca_3.1 protein band signal with that of the ACTB signal, the K_Ca_3.1 signal in “normal” was expressed as 1.0 (*n* = 4 for each). (**D**) Voltage-sensitive fluorescent dye imaging of 1 μM TRAM-34-induced depolarization responses in the splenic CD4^+^ T cells of normal and IBD model mice. Cells were isolated from three different mice in each group. The cell numbers that were used in the experiments are shown in parentheses. The fluorescent intensity of DiBAC_4_(3) before the application of TRAM-34 at 0 s is expressed as 1.0. Images were measured every 5 s. (**E**) Summarized data are shown as 1 µM TRAM-34-induced depolarization responses [∆ relative fluorescence intensity of DiBAC_4_(3)] in normal and IBD model mice. The values of fluorescent intensity were determined by measuring the average for 1 min (12 images) before quitting the application of 1 µM TRAM-34. The results are expressed as means ± SEM. *, **: *p* < 0.05, 0.01 vs. normal mice (normal).

We measured the expression and activity of K_Ca_3.1 in the splenic CD4^+^ T cells of normal and IBD model mice using the real-time PCR assay, Western blotting, and voltage-sensitive dye imaging. Similar to our previous study [8], a significant increase was observed in the expression level of K_Ca_3.1 transcripts and proteins in the CD4^+^CD25^−^ T cells of IBD model mice (Figure 1A,C). The expression levels of the K_Ca_3.1 transcripts that were relative to β-actin (ACTB) (in arbitrary units) were 0.028 ± 0.001 and 0.053 ± 0.003 in normal and IBD model mice, respectively (*n* = 4 for each, *p* < 0.01) (Figure 1A), and those of the K_Ca_3.1 proteins were approximately 1.8-fold higher in IBD model mice compared with the normal ones (*n* = 4 for each, *p* < 0.01) (Figure 1C). Among six K_Ca_3.1 function-modifying molecules (NDPK-B, PI3KC2B, PHPT1, MTMR6, TRIM-27, and PGAM5), the positive regulator of K_Ca_3.1 activity, NDPK-B was the most abundantly expressed in splenic CD4^+^CD25^−^ T cells, and a significant increase was observed in its expression level (Figure 1B). The expression levels of NDPK-B in arbitrary units were 0.076 ± 0.001 and 0.126 ± 0.010 in normal and IBD model mice, respectively (*n* = 4 for each, *p* < 0.05). The expression levels of two negative regulators of K_Ca_3.1 activity, MTMR6 and TRIM-27, were significantly increased, whereas no significant changes were noted in the other regulators (Figure S1A–E). The up-regulation of the inflammatory cytokines IFN-γ and IL-17A was observed in the CD4^+^CD25^−^ T cells of IBD model mice (Figure S1F,G). Concomitant with the up-regulation of K_Ca_3.1 and NDPK-B, TRAM-34 (1 μM)-induced depolarization responses in CD4^+^ T cells were significantly stronger in IBD model mice than in the normal group (*n* = 17 and 10, *p* < 0.01) (Figure 1D,E).

**Figure 2:**
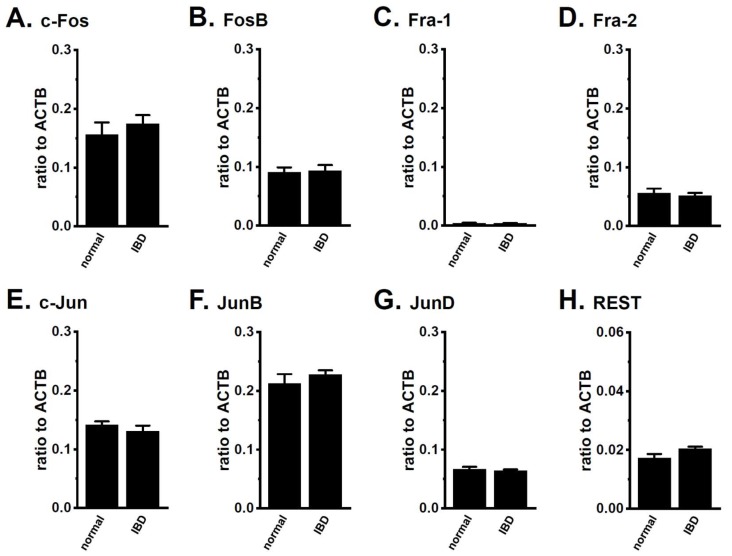
No significant changes in expression levels of AP-1 and REST transcripts in splenic CD4^+^ T cells of IBD model mice. Real-time PCR assay for AP-1 components [c-Fos (**A**), FosB (**B**), Fra-1 (**C**), Fra-2 (**D**), c-Jun (**E**), JunB (**F**), and JunD (**G**)] and REST (**H**) in the splenic CD4^+^ T cells of “normal” and “IBD” model mice (*n* = 4 for each). Expression levels were expressed as a ratio to ACTB. The results are expressed as means ± SEM.

**Figure 3:**
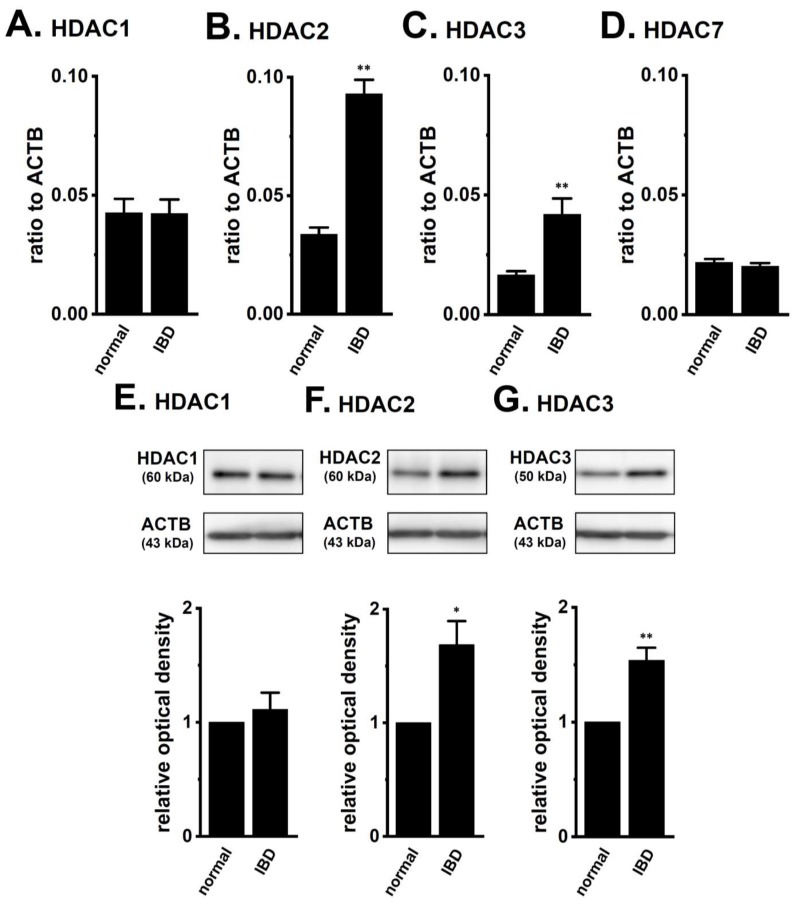
Expression levels of HDAC1, 2, 3, and 7 in splenic CD4^+^ T cells of IBD model mice. (**A**–**D**) Real-time PCR assay for HDAC1 (**A**), HDAC2 (**B**), HDAC3 (**C**), and HDAC7 (**D**) in the splenic CD4^+^ T cells of “normal” and “IBD” model mice (*n* = 4 for each). Expression levels were expressed as a ratio to ACTB. (**E**–**G**) Protein lysates of the splenic CD4^+^ T cells of “normal” and “IBD” model mice were probed by immunoblotting with anti-HDAC1 (**E**), HDAC2 (**F**), HDAC3 (**G**) (upper panel) and anti-ACTB (lower panel, **E**–**G**) antibodies on the same filter. Summarized results were obtained as the optical density of HDACs and ACTB band signals. After compensation for the optical density of the HDAC protein band signal with that of the ACTB signal, the HDAC signal in the vehicle control was expressed as 1.0 (*n* = 4 for each). The results are expressed as means ± SEM. *, **: *p* < 0.05, 0.01 vs. normal mice.

AP-1 (Fos/Jun homo-/hetero-dimer), REST, and HDACs are transcriptional and post-transcriptional regulators of K_Ca_3.1 [15,16,17,18,19,20,21]. As shown in Figure 2, no significant changes were detected in the expression levels of the Fos family (c-Fos, FosB, Fra-1, and Fra-2) (Figure 2A–D), Jun family (c-Jun, JunB, and JunD) (Figure 2E–G), and REST (Figure 2H) transcripts in the CD4^+^CD25^−^ T cells of the IBD model mice. We subsequently compared the expression levels of HDACs between the CD4^+^CD25^−^ T cells of normal and IBD model mice. Among the eleven isoforms that were examined, relatively high expression levels of HDAC1, HDAC2, HDAC3, and HDAC7 were found in CD4^+^CD25^−^ T cells, and a significant increase in the expression levels of HDAC2 and HDAC3 was noted in IBD model mice (Figure 3A–D). The expression levels of HDAC2 and HDAC3 transcripts in arbitrary units were 0.034 ± 0.003 and 0.093 ± 0.006 (for HDAC2) and 0.017 ± 0.002 and 0.042 ± 0.007 (for HDAC3) in the CD4^+^CD25^−^ T cells of normal and IBD model mice, respectively (*n* = 4 for each, *p* < 0.01) (Figure 3B,C). Correspondingly, the expression levels of HDAC2 and HDAC3 proteins were approximately 1.7 (for HDAC2)- and 1.5 (for HDAC3)-fold higher in IBD model mice compared with normal ones (Figure 3F,G), and no significant change was observed in the expression levels of HDAC1 proteins in IBD model mice (Figure 3E). The other HDAC isoforms were less abundantly expressed in CD4^+^CD25^−^ T cells, and no significant changes in their expression was found in the IBD model mice (Figure S2). These results are consistent with previous findings that were reported by Felice et al. (2015) [28], showing that HDAC2 and HDAC3 (also HDAC6, HDAC9, and HDAC10) isoforms were involved in chronic intestinal inflammation.

### 2.2. Decreased Expression and Activity of K_Ca_3.1 by Treatment with the Pan-HDACi, Vorinostat, and HDAC2- and HDAC3-Selective HDACis in CD4^+^ T Cells of IBD Model Mice

**Figure 4:**
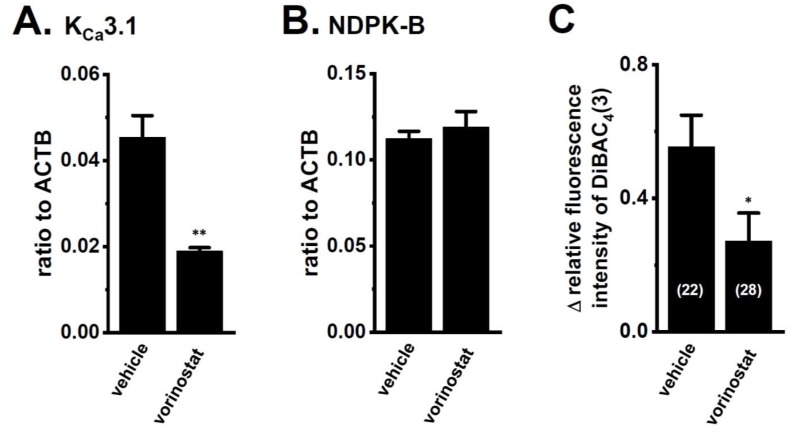
Decrease in the gene expression and activity of K_Ca_3.1 by the treatment with 1 μM vorinostat for 24 h in splenic CD4^+^ T cells of IBD model mice. (**A**,**B**) Real-time PCR assay for K_Ca_3.1 (**A**) and NDPK-B (**B**) in “vehicle”- and “vorinostat”-treated CD4^+^ T cells (*n* = 4 for each). Expression levels were expressed as a ratio to ACTB. (**C**) Summarized data are shown as 1 μM TRAM-34-induced depolarization responses in vehicle- and vorinostat-treated CD4^+^ T cells. Cells were isolated from three different mice in each group. Cell numbers that were used for the experiments are shown in parentheses. The results are expressed as means ± SEM. *, **: *p* < 0.05, 0.01 vs. vehicle control.

In order to investigate the involvement of HDACs in K_Ca_3.1 transcription in the CD4^+^ T cells of IBD model mice, we examined the effects of the pan-HDAC inhibitor, vorinostat on the expression and activity of K_Ca_3.1 in the CD4^+^ T cells of IBD model mice. A total of 5 μg/mL Con A and 10 U/mL IL-2 were added as culture medium supplements to maintain viable and healthy lymphocytes during cultivation [29,30]. No significant changes in the expression levels of the K_Ca_3.1, HDAC2, or HDAC3 transcripts were found 48 h after T-cell activation (*n* = 4 for each, *p* > 0.05 vs. 0 h) (Figure S3). CD4^+^ T cells were treated with 1 μM vorinostat for 24 h. A significant decrease in the expression level of K_Ca_3.1 was found in the CD4^+^CD25^−^ T cells of the vorinostat-treated group (Figure 4A). The expression levels of K_Ca_3.1 in arbitrary units were 0.045 ± 0.005 and 0.019 ± 0.001 in the vehicle- and vorinostat-treated groups, respectively (*n* = 4 for each, *p* < 0.01 vs. vehicle control). No significant changes in the expression levels of NDPK-B or the other K_Ca_3.1 function-modifying molecule transcripts were found in the vorinostat-treated group (*n* = 4 for each, *p* > 0.05) (Figure 4B and Figure S4). Accordingly, TRAM-34-induced depolarization responses were significantly reduced by the treatment with vorinostat (*n* = 22 and 28, *p* < 0.05) (Figure 4C).

**Figure 5:**
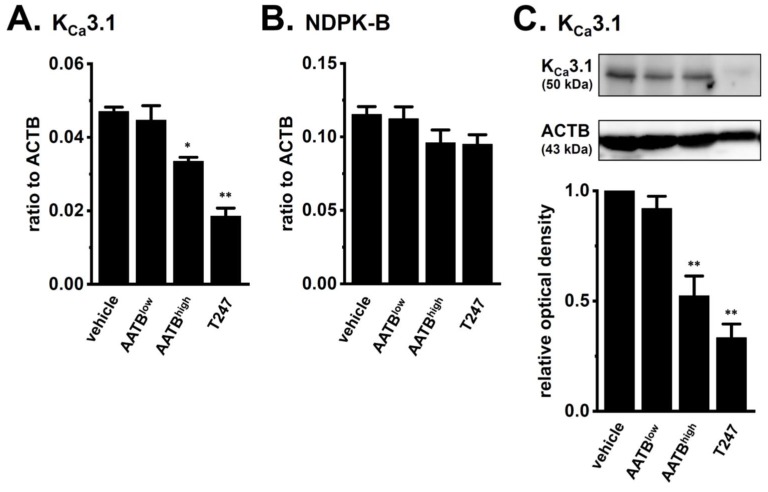
Effects of the treatment with the selective HDACis, AATB and T247 on the expression of K_Ca_3.1 and NDPK-B in splenic CD4^+^ T cells of IBD model mice. (**A**,**B**) Real-time PCR assay for K_Ca_3.1 (**A**) and NDPK-B (**B**) in “vehicle”-, 30 nM AATB (“AATB^low^”)-, 300 nM AATB (“AATB^high^”)-, and 1 μM “T247”-treated CD4^+^ T cells (*n* = 4 for each). Expression levels were expressed as a ratio to ACTB. (**C**) Protein lysates of in vehicle-, AATB^low^-, AATB^high^-, and T247-treated CD4^+^ T cells were probed by immunoblotting with anti-K_Ca_3.1 (upper panel) and anti-ACTB (lower panel) antibodies on the same filter. Summarized results were obtained as the optical density of K_Ca_3.1 and ACTB band signals. After compensation for the optical density of the K_Ca_3.1 protein band signal with that of the ACTB signal, the K_Ca_3.1 signal in the vehicle control was expressed as 1.0 (*n* = 4 for each). Results are expressed as means ± SEM. *, **: *p* < 0.05, 0.01 vs. vehicle control.

We also examined the effects of selective HDACis on the expression level of K_Ca_3.1 in the CD4^+^CD25^−^ T cells of IBD model mice: AATB, the half maximal inhibitory concentration (IC_50_) = 0.007 and 0.049 μM for HDAC1 and HDAC2, respectively and T247, IC_50_ = 0.24 μM for HDAC3 [31,32]. Similar to our previous study [33], 30 nM AATB (AATB^low^), 300 nM AATB (AATB^high^), and 1 µM T247 were used as HDAC1, HDAC1/2, and HDAC3-selective HDACis, respectively, in the present study. As shown in Figure 5A, a significant decrease in the expression level of K_Ca_3.1 transcripts was found following the treatment with 300 nM AATB (*n* = 4 for each, *p* < 0.05 vs. vehicle control) and 1 μM T247 (*n* = 4 for each, *p* < 0.01), but not 30 nM AATB (*n* = 4 for each, *p* > 0.05). Correspondingly, a significant decrease in the expression level of K_Ca_3.1 proteins was found following the treatment with 300 nM AATB (*n* = 4 for each, *p* < 0.01 vs. vehicle control) and 1 μM T247 (*n* = 4 for each, *p* < 0.01) (Figure 5C). Approximately a 55% decrease in the expression levels of K_Ca_3.1 proteins were found following the treatment with 1 µM vorinostat. No significant changes were observed in the expression levels of NDPK-B or the other K_Ca_3.1 function-modifying molecule transcripts in the HDACi-treated groups (*n* = 4 for each, *p* > 0.05) (Figure 5B and Figure S4). These results suggest that the up-regulation of HDAC2 and HDAC3 contributed to the increases that were observed in the expression and activity of K_Ca_3.1 in the inflammatory CD4^+^ T cells of IBD model mice.

### 2.3. Decreased Expression Levels of K_Ca_3.1 by the Inhibition of HDAC2 and HDAC3 in Con A-Stimulated Mouse Thymocytes

**Figure 6:**
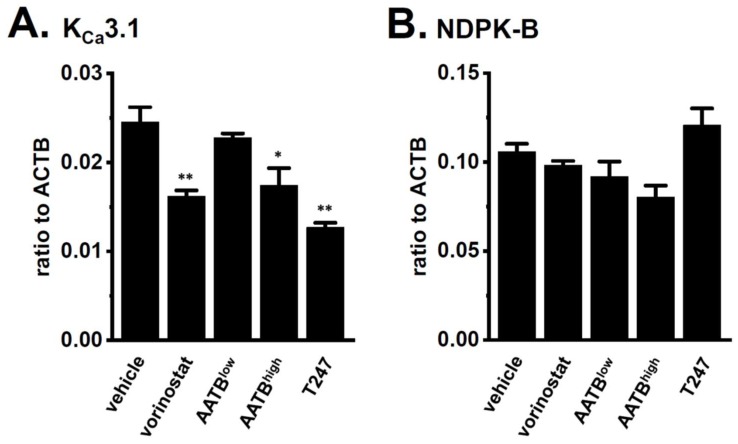
Effects of a treatment with HDACis for 24 h on the gene expression of K_Ca_3.1 and NDPK-B in mouse thymocytes stimulated by concanavalin A (5 µg/mL). (**A**,**B**) Real-time PCR assay for K_Ca_3.1 (**A**) and NDPK-B (**B**) in “vehicle”-, 1 μM “vorinostat”-, 30 nM AATB (“AATB^low^”)-, 300 nM AATB (“AATB^high^”)-, and 1 μM “T247”-treated mouse thymocytes (*n* = 4 for each). Expression levels were expressed as a ratio to ACTB. The results are expressed as means ± SEM. *, **: *p* < 0.05, 0.01 vs. vehicle control.

**Figure 7:**
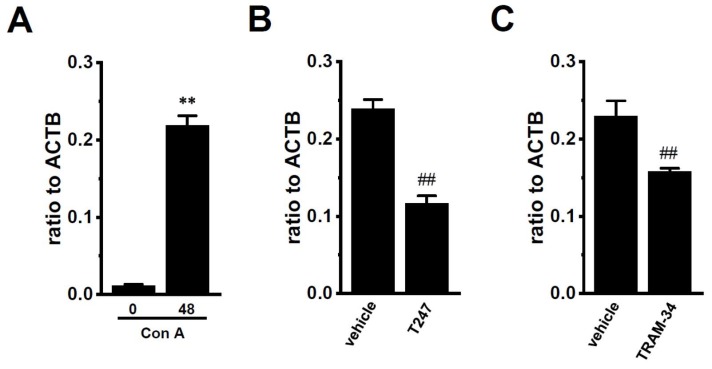
Effects of treatments with 1 µM T247 for 24 h and 1 µM TRAM-34 for 12 h on the gene expression of CD25 in Con A-stimulated mouse thymocytes. (**A**) Real-time PCR assay for CD25 in Con A-stimulated mouse thymocytes for 0 or 48 h. (**B**,**C**) Real-time PCR assay for CD25 in Con A-stimulated mouse thymocytes that were treated with 1 µM T247 for 24 h (**B**) and 1 µM TRAM-34 for 12 h (**C**). Expression levels were expressed as a ratio to ACTB. The results are expressed as means ± SEM. **: *p* < 0.01 vs. Con A-stimulated for 0 h. ^##^: *p* < 0.01 vs. vehicle control.

In thymus-derived T_reg_-like cells that were induced by the Con A stimulation, the treatments with vorinostat, AATB^high^, and T247 significantly decreased the expression levels of K_Ca_3.1 (*n* = 4 for each, *p* < 0.01 in vorinostat-treated, *p* < 0.05 in AATB^high^-treated, and *p* < 0.01 in T247-treated), but not for NDPK-B (*n* = 4 for each, *p* > 0.05 in both) (Figure 6). Wang et al. (2015) showed that HDAC3 plays an important role in the development and function of thymus-derived regulatory T (T_reg_) cells [27]. Mouse thymocytes stimulated by Con A showed the up-regulation of a marker molecule for T_reg_ cells (CD25) (Figure 7A). The expression levels of CD25 were decreased by the treatment with T247 for 24 h (*n* = 4 for each, *p* < 0.01) (Figure 7B). Both expression levels were also significantly decreased by the treatment of Con A-stimulated thymocytes with the K_Ca_3.1 blocker, 1 µM TRAM-34 for 12 h (*n* = 4 for each, *p* < 0.01) (Figure 7C). Previous studies showed that K_Ca_3.1 is an essential contributor to T cell differentiation [1,5]. These findings suggest that the HDAC3-mediated functional regulation of K_Ca_3.1 is involved in the development and function of T_reg_ cells.

## 3. Discussion

IBD, including ulcerative colitis (UC) and CD, are characterized by chronic inflammation of the colon. We previously reported that the up-regulated expression of the Ca^2+^-activated K^+^ channel K_Ca_3.1 in inflammatory CD4^+^ T cells was involved in the pathogenesis of IBD [4]. Similarly, the expression levels of K_Ca_3.1 transcripts were higher in surgical specimens and endoscopic biopsies from IBD patients [9]. However, the underlying molecular mechanisms have not yet been elucidated. Recent studies implicated particular HDAC isoforms, HDAC2, HDAC3, HDAC6, HDAC9, and HDAC10, in chronic intestinal inflammation and, thus, selective HDACis serve as attractive therapeutic options for IBD [22,25,28,34,35]. The main results of the present study are as follows: (1) significant increases in HDAC2 and HDAC3 (Figure 3) and (2) the down-regulation of K_Ca_3.1 by the pharmacological inhibition of HDAC2 or HDAC3 in the inflammatory CD4^+^ cells of IBD model mice (Figure 5). Similar epigenetic modifications to K_Ca_3.1 were found in the “human” T cell model, the T-cell lymphoma cell line HuT-78 (unpublished data). T cells also express the other K^+^ channels: voltage-gated K^+^ channel K_V_1.3 and two-pore-domain K^+^ channel K_2P_5.1 [1,2,3,4]. No significant changes in the expression levels of K_V_1.3 and K_2P_5.1 transcripts following treatment with HDACis were detected in the CD4^+^ T cells of IBD model mice (Figure S5).

AP-1, a homo- and hetero-dimeric transcription factor composed of members of the Fos/Jun families, regulates K_Ca_3.1 transcription in T cells [15]. Moriyama et al. reported that intestinal inflammation in IBD model mice was attenuated by the knockdown of AP-1 [36]. Previous studies demonstrated (1) the up-regulation of JunB (approximately 1.5-fold) in colon biopsies from CD patients [37]; (2) the up-regulation of Fra-1 in bone from IBD model rats [38]; and (3) the up-regulation of c-Fos, but not c-Jun or JunB in the colons of UC patients [39]. In the present study, c-Fos, FosB, c-Jun, and JunB were expressed at relatively high levels in the CD4^+^CD25^−^ T cells of normal mice, whereas no significant changes were observed in the expression levels of the AP-1 member transcripts that were examined in IBD model mice (Figure 2A–G). The down-regulation of REST and REST co-repressors may result in the up-regulation of K_Ca_3.1 in inflammatory CD4^+^ T cells. Costello et al. reported the up-regulation of the REST co-repressor 3 (approximately 3-fold) in colon biopsies from CD patients [37] and no significant changes in the expression levels of the REST transcripts that were examined in IBD model mice (Figure 2H). These results suggest that AP-1 and REST do not contribute to the up-regulated expression of K_Ca_3.1 in the inflammatory CD4^+^ cells of IBD model mice (Figure 1).

We previously reported epigenetic modifications to K_Ca_3.1 by HDAC2 and HDAC3 in human breast cancer cells [21]. Similarly, in the splenic CD4^+^CD25^−^ T cells of IBD model mice, the expression levels of HDAC2 and HDAC3 isoforms were specifically up-regulated (Figure 3B,C), and the pharmacological inhibition of HDAC2 or HDAC3 resulted in the significant down-regulation of K_Ca_3.1 (Figure 5). NDPK-B expression was also up-regulated in the CD4^+^CD25^−^ T cells of IBD model mice, however this was not affected by treatment with HDACis (Figure 4B and Figure 5B). These results indicate that HDAC2 and HDAC3 regulate K_Ca_3.1 expression and activity in T cells. NDPK-B activates K_Ca_3.1 by histidine phosphorylation in the C terminus of it [10]. In the present study, the expression levels of NDPK-B were significantly increased in CD4^+^ T cells of IBD model (Figure 1B), suggesting that NDPK-B upregulation may also be involved in the increased K_Ca_3.1 activity in the CD4^+^ T cells of IBD model mice. However, it was not affected by treatment with HDACis (Figure 4B and Figure 5B). Further studies are needed to clarify the mechanisms that are responsible for the up-regulated expression of NDPK-B in the IBD model. In addition to HDAC2 and HDAC3, HDAC6 and HDAC9 are also associated with chronic intestinal inflammation [28]. The selective inhibition of HDAC6 suppressed CD19^+^ B cell infiltration into the inflamed colonic lamina propria in DSS-induced IBD model mice [40]. HDAC9 plays an important role in T_reg_ function, and HDAC9 knockout mice are resistant to the pathogenesis of IBD [25]. As shown in Figure S2C,E, the less abundant expression of HDAC6 and HDAC9 was observed in CD4^+^CD25^−^ T cells. However, our preliminary study showed extremely low HDAC9 expression levels in the CD4^+^CD25^+^ T cells of normal and IBD model mice (less than 0.001 in arbitrary units, with two different primer pairs). Therefore, the contribution of HDAC9 to K_Ca_3.1 transcription in mouse splenic CD4^+^CD25^+^ cells and Con A-stimulated thymocytes was not investigated in the present study. Wang et al. (2015) showed that HDAC3 plays an important role in the development and function of thymus-derived as well as induced T_reg_ cells [27]. As shown in Figure 7, the up-regulated key marker of T_reg_ cells, CD25, was significantly decreased by the pharmacological inhibition of HDAC3, and similar results were obtained following the pharmacological inhibition of K_Ca_3.1. These results suggest that the HDAC3-mediated functional regulation of K_Ca_3.1 is involved in the development and function of T_reg_ cells. However, in the acute IBD model that was used in the present study, no significant changes were found in the expression levels of K_Ca_3.1 or HDAC3 in splenic CD4^+^CD25^+^ cells (Figure S6). T_reg_ cells decreased during the active stage of IBD and increased during the remission period at the chronic stage of IBD [41]. Further studies are needed to elucidate the pathophysiological significance of HDAC3-mediated K_Ca_3.1 regulation in the development and function of T_reg_ cells using chronic IBD models.

K_Ca_3.1 is a potential therapeutic target for not only IBD, but also for other autoimmune disorders, asthma, atherosclerosis, and fibrosis [2,4]. Similarly, HDAC2 and HDAC3 are key regulators of the following disorders: rheumatoid arthritis [42], severe asthma and chronic obstructive pulmonary disease [43,44,45], and atherosclerosis [46,47]. The contribution of HDAC2 to tissue fibrosis is attracting increasing attention [48,49]. Recent studies showed that K_Ca_3.1 inhibitors are a potential treatment option for renal, liver, kidney, corneal, and pulmonary fibrosis [50,51,52,53,54]. It currently remains unclear whether epigenetic modifications by HDAC2/3 are involved in these K_Ca_3.1-related disorders; therefore, further studies are needed to clarify the therapeutic effectiveness of selective HDACis in various K_Ca_3.1-related disorders.

## 4. Materials and Methods

### 4.1. Preparation of a DSS-Induced Mouse IBD Model and Isolation of CD4^+^ T Cells Using Dynabeads

Male C57BL/6J (5–6 weeks of age) (Figure 1, Figure 2, Figure 3, Figure 4 and Figure 5) and Balb/c (3–4 weeks of age) (Figure 6 and Figure 7) mice were purchased from Japan SLC (Shizuoka, Japan) and were acclimatized for 1 week before the experiment. They were given distilled water containing 5% (*wt*/*vol*) dextran sulfate sodium 5000 (DSS) (Wako Pure Chemical, Osaka, Japan) *ad libitum*. The control mice were given drinking water only. Seven days after the administration of DSS, the mice were euthanized, the spleens were isolated, and colitis and inflammation were assessed, as described in our previous study [8]. All experiments were performed in accordance with the Guiding Principles for the Care and Use of Laboratory Animals in Nagoya City University (NCU) and Kyoto Pharmaceutical University (KPU), and also with the approval of the presidents of both universities (NCU, No. H29M-50, 5 October 2017; KPU, No. 17-034, 1 April 2017). Single-cell suspensions were prepared by pressing the spleen with a frosted slide grass and then filtering through cell strainers. CD4^+^CD25^−^ (for the real-time PCR assay) or CD4^+^ (for fluorescent voltage-sensitive dye imaging) T cells were isolated from splenic cell suspensions using the Dynabeads FlowComp mouse CD4^+^ kit or Dynabeads FlowComp mouse CD4^+^CD25^+^ Treg cell kit according to the experimental manual supplied by Thermo Fisher Scientific (Waltham, MA, USA). Flow cytometric analyses confirmed that 95% of the purified T cells were CD4^+^CD25^−^ or CD4^+^.

### 4.2. RNA Extraction, Reverse Transcription (RT)-PCR, and Real-Time PCR

Total RNA extraction and RT-PCR from mouse splenic CD4^+^CD25^−^ T lymphocytes were performed as previously reported [8]. The resulting cDNA products were amplified with gene-specific primers and primers that were designated by the use of Primer Express software (ver. 3.0.1, Thermo Fisher Scientific). Quantitative real-time PCR was performed with the use of Sybr Green chemistry (SYBR Premix Ex Taq II) (TaKaRa BIO, Osaka, Japan) on the ABI 7500 fast real-time PCR instrument (Thermo Fisher Scientific), as previously reported [8]. The following PCR primers for mouse clones were used for real-time PCR: K_Ca_3.1 (GenBank accession number: NM_008433, 343–452), amplicon = 110 bp; NDPK-B (NM_008705, 467–597), 131 bp; HDAC1 (NM_008228, 824–944), 121 bp; HDAC2 (NM_008229, 1436–1546), 111 bp; HDAC3 (NM_010411, 1106–1226), 121 bp; HDAC7 (NM_001204275, 2611–2732), 122 bp; c-Fos (NM_010234, 581–691), 111 bp; FosB (NM_008036, 1044–1173), 130 bp; Fra-1 (NM_010235, 855–986), 132 bp; Fra-2 (NM_008037, 331–457), 127 bp; c-Jun (NM_010591, 1792–1921), 130 bp; JunB (NM_008416, 1169–1309), 141 bp; JunD (NM_010592, 2185–2295), 111 bp; REST (NM_011263, 1022–1153), 132 bp; CD25 (NM_000417, 750–869), 120 bp; ACTB (NM_007393, 418–518), 101 bp. Regression analyses of the mean values of three multiplex RT-PCRs for log_10_-diluted cDNA were used to generate standard curves. Unknown quantities relative to the standard curve for a particular set of primers were calculated, yielding the transcriptional quantitation of gene products relative to the endogenous standard, ACTB [8].

### 4.3. Western Blotting

Protein lysates were prepared from mouse CD4^+^ T cells using RIPA lysis buffer for Western blotting. After the quantification of protein concentrations using the BIO-RAD DC^TM^ protein assay, protein lysates were subjected to SDS-PAGE (10%). Blots were incubated with anti-HDAC1 (H-51) (Santa Cruz Biotechnology, Santa Cruz, CA, USA), anti-HDAC2 (H-54) (Santa Cruz Biotechnology), anti-HDAC3 (H-99) (Santa Cruz Biotechnology), anti-K_Ca_3.1 (Alomone Labs, Jerusalem, Israel), and anti-ACTB (Medical & Biological Laboratories, Nagoya, Japan) antibodies, and were then incubated with anti-rabbit horseradish peroxidase-conjugated IgG (Merck, Darmstadt, Germany). An enhanced chemiluminescence detection system (Nacalai Tesque, Kyoto, Japan) was used to detect the bound antibody. The resulting images were analyzed using Amersham Imager 600 (GE Healthcare Japan, Tokyo, Japan). The light intensities of the band signals relative to that of the ACTB signal were calculated using ImageJ software (Ver. 1.42, NIH, Bethesda, MA, USA). In the summarized results, relative protein expression levels in the control were expressed as 1.0.

### 4.4. Measurement of Membrane Potentials Using Fluorescent Voltage-Sensitive Dyes

Isolated splenic CD4^+^ T cells were cultivated in RPMI 1640 medium that was supplemented with 10% heat-inactivated fetal calf serum (Merck, Darmstadt, Germany), antibiotics (0.1% penicillin and 0.1% streptomycin), concanavalin A (Con A, 5 µg/mL), and IL-2 (10 U/mL) for 48 h. Membrane potentials were measured using the voltage-sensitive dye bis-(1,3-dibutylbarbituric acid)trimethine oxonol [DiBAC_4_(3)], as previously reported [8]. Changes induced in the fluorescent intensity of DiBAC_4_(3) by 1 µM TRAM-34 were measured using an ORCA-Flash2.8 digital camera (Hamamatsu Photonics, Hamamatsu, Japan). Data collection and analyses were performed using an HCImage system (Hamamatsu Photonics). Images were measured every 5 s.

### 4.5. Chemicals

The sources of pharmacological agents were as follows: DiBAC_4_(3) (Dojindo, Kumamoto, Japan) and TRAM-34 (1-[(2-Chlorophenyl)diphenylmethyl]-1H-pyrazole) (Toronto Research Chemicals, Toronto, ON, Canada). The selective HDACis AATB, 4-(acetylamino)-*N*-[2-amino-5-(2-thienyl)phenyl]-benzamide and T247, *N*-(2-aminophenyl)-4-[1-(2-thiophen-3-ylethyl)-1*H*-[1],[2],[3]triazol-4-yl]benzamide were supplied by Professor Suzuki (Kyoto Prefectural University of Medicine, Kyoto, Japan). All other agents were obtained from Merck or Wako Pure Chemical Industries (Tokyo, Japan).

### 4.6. Statistical Analysis

The significance of differences between two and among multiple groups was evaluated by the Student’s *t*-test, Welch’s *t*-test, or Tukey’s test after the *F* test or ANOVA. Results with a *p*-value of less than 0.05 or 0.01 were considered to be significant. The data were presented as means ± SEM.

## 5. Conclusions

Epigenetic modifications are associated with channelopathies, and the potential of HDACis as therapeutic drugs for autoimmune and inflammatory disorders has recently been proposed [55]. The present study demonstrated that epigenetic modifications by histone deacetylases (HDAC2 and HDAC3) play, at least in part, an important role in the up-regulation of K_Ca_3.1 and its increased activity in CD4^+^ T cells, resulting in enhanced inflammatory cytokine production and T cell activation, proliferation, and differentiation. K_Ca_3.1 is a potential therapeutic target for autoimmunity, asthma, atherosclerosis, and fibrosis. The present results provide valuable insights into these K_Ca_3.1-related immune disorders.

## Supplementary Materials

Supplementary materials can be found at http://www.mdpi.com/1422-0067/19/10/2942/s1.
