# Supplementary material for: Histone Deacetylases Enhance Ca2+-Activated K+ Channel KCa3.1 Expression in Murine Inflammatory CD4+ T Cells
Source: Int J Mol Sci. 2018 Sep 27;19(10):2942. doi: 10.3390/ijms19102942 (PMC6213394; doi:10.3390/ijms19102942)
Supplement: Supplementary file 1 [file ijms-19-02942-s001.pdf]

Supplementary Materials: Histone Deacetylases Enhance Ca^{2+}-Activated K^+ Channel $K_{Ca}3.1$ Expression in Murine Inflammatory $CD4^+$ T Cells

Miki Matsui, Kyoko Terasawa, Junko Kajikuri, Hiroaki Kito, Kyoko Endo, Pattaporn Jaikhan, Takayoshi Suzuki and Susumu Ohya

In supplementary Figures, the following PCR primers for mouse clones were used for real-time PCR: PI3K-C2B (NM_001099276, 2293–2422), 130 bp; PHPT-1 (NM_029293, 69–189), 121 bp; MTMR-6 (NM_144843, 722–832), 111 bp; TRIM-27 (NM_009054, 1530–1650), 121 bp; PGAM5 (NM_001163538, 341–460), 120 bp; HDAC4 (NM_207225, 2833–2954), 122 bp; HDAC5 (NM_001077696, 2741–2862), 122 bp; HDAC6 (NM_010413, 3231–3351), 121 bp; HDAC8 (NM_027382, 969–1069), 101 bp; HDAC9 (NM_001271386, 1093–1212/1960–2080), 120/121 bp; HDAC10 (NM_199198, 1702–1822), 121 bp; HDAC11 (NM_144919, 531–652), 122 bp; IFN-γ (NM_008337, 222–323), 102 bp; IL-17A (NM_010552, 165–277), 113 bp; $K_V1.3$ (NM_008418, 902–1063), 160 bp; $K_{2P}5.1$ (NM_001650, 1560–1680), 121 bp.

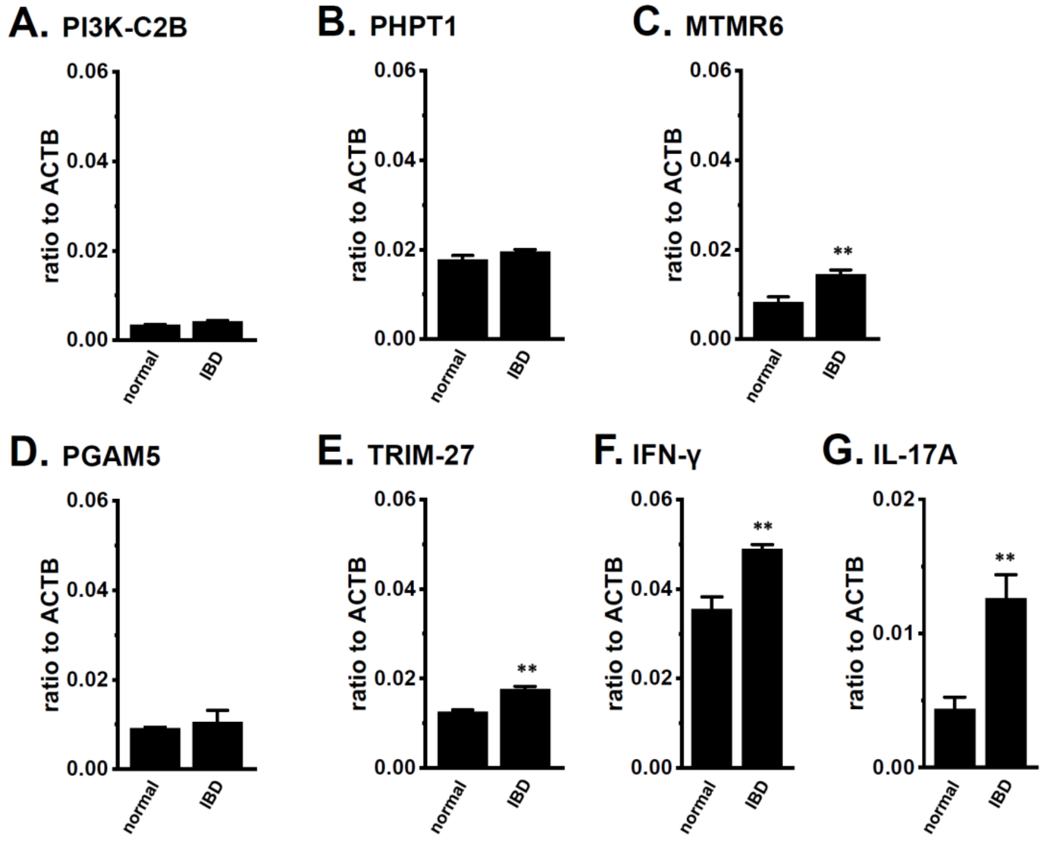

Figure S1. Gene expression of $K_{Ca}3.1$ function-modifying molecules, excluding NDPK-B and inflammatory cytokines, in the splenic $CD4^+$ T cells of normal and IBD model mice. (**A**–**E**): Real-time PCR assay for PI3K-C2B (**A**), PHPT-1 (**B**), MTMR6 (**C**), PGAM5 (**D**), TRIM-27 (**E**), IFN-γ (**F**), and IL-17A (**G**) in the splenic $CD4^+$ T cells of 'normal' and 'IBD' model mice ($n = 4$ for each). Expression levels were expressed as a ratio to ACTB. Results are expressed as means ± SEM. **: $p < 0.01$ vs. normal mice.

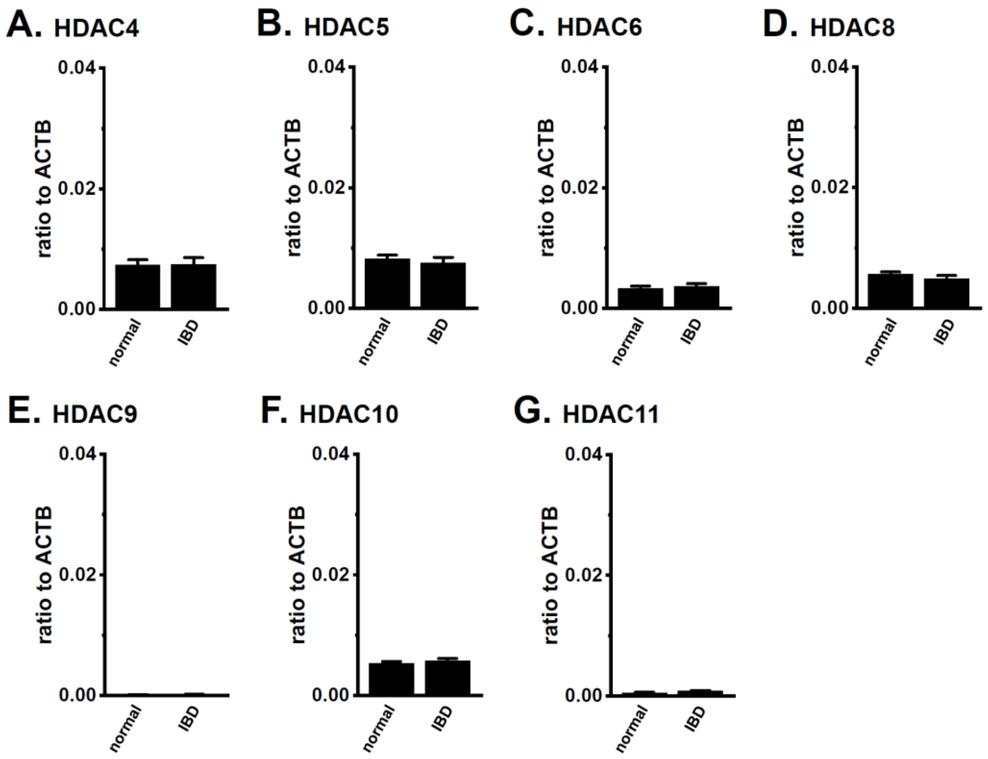

Figure S2. Gene expression of HDAC isoforms (HDAC4-6 and HDAC8-11) in splenic CD4$^+$ T cells of normal and IBD model mice. A-G: Real-time PCR assay for HDAC4 (**A**), HDAC5 (**B**), HDAC6 (**C**), HDAC8 (**D**), HDAC9 (**E**), HDAC10 (**F**), and HDAC11 (**G**) in the splenic CD4$^+$ T cells of "normal" and "IBD" model mice (n = 4 for each). Expression levels were expressed as a ratio to ACTB. Results are expressed as means ± SEM.

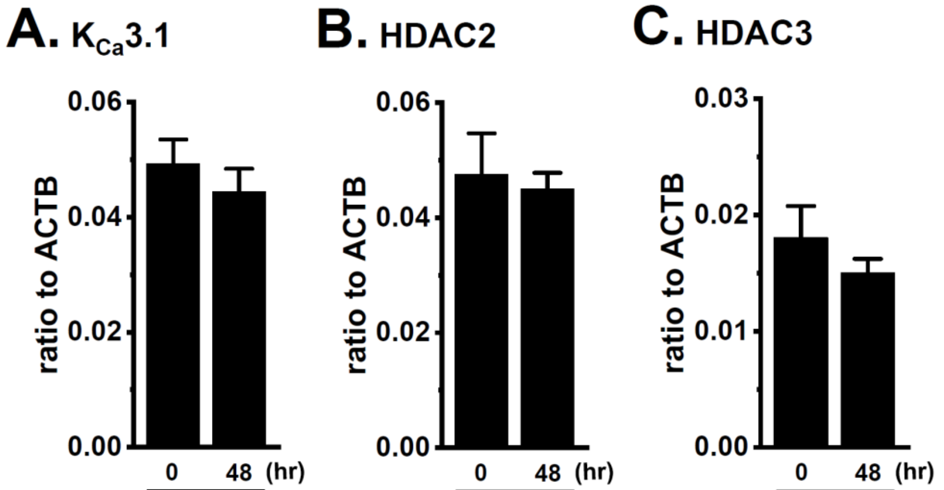

Figure S3. No significant changes in expression levels of K_{Ca}3.1, HDAC2, and HDAC3 transcripts by the concanavalin A (Con A) treatment for 48 h in normal mouse thymocytes. (**A**–**C**): Real-time PCR assay for K_{Ca}3.1 (**A**), HDAC2 (**B**), and HDAC3 (**C**) in 5 μg/mL Con A-treated mouse thymocytes for 0 and 48 h (n = 4 for each). Expression levels were expressed as a ratio to ACTB. Results are expressed as means ± SEM.

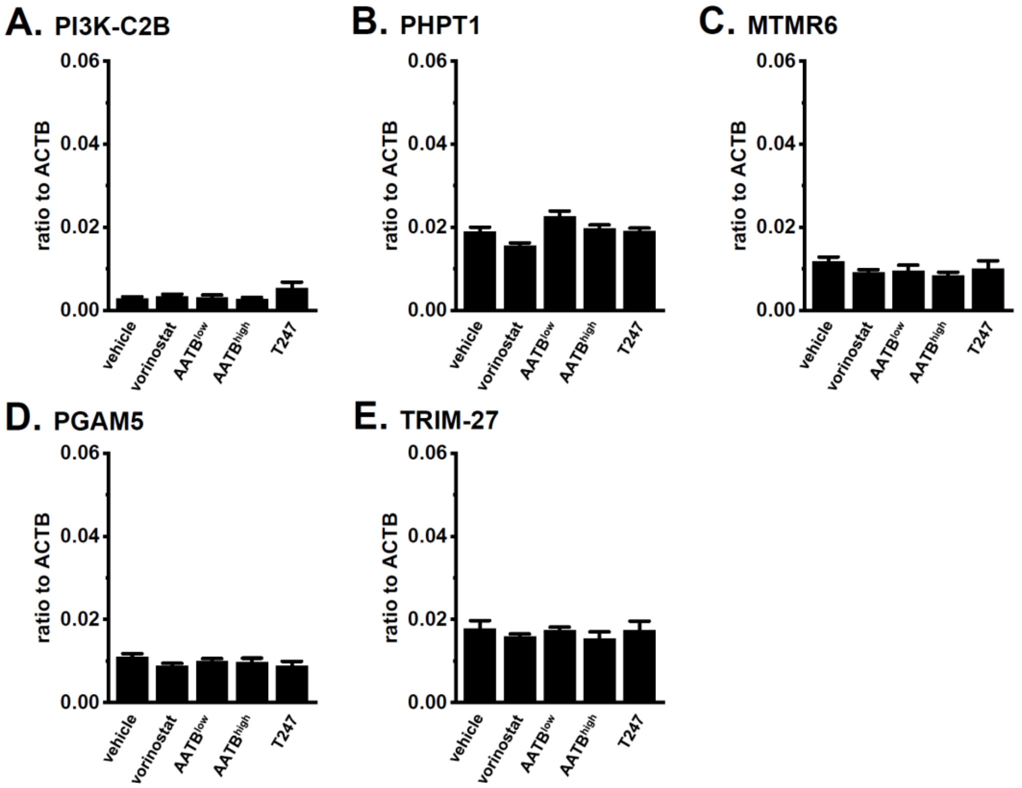

Figure S4. Effects of HDACis on the gene expression of $K_{Ca}3.1$ function-modifying molecules in splenic $CD4^+$ T cells of IBD model mice. A-E: Real-time PCR assay for PI3K-C2B (**A**), PHPT-1 (**B**), MTMR6 (**C**), PGAM5 (**D**), and TRIM-27 (**E**) in "vehicle"-, 1 μM "vorinostat"-, 30 nM AATB ("AATB^{low}")-, 300 nM AATB ("AATB^{high}")-, and 1 μM "T247"-treated splenic $CD4^+$ T cells of IBD model mice (*n* = 4 for each). Expression levels were expressed as a ratio to ACTB. Results are expressed as means ± SEM.

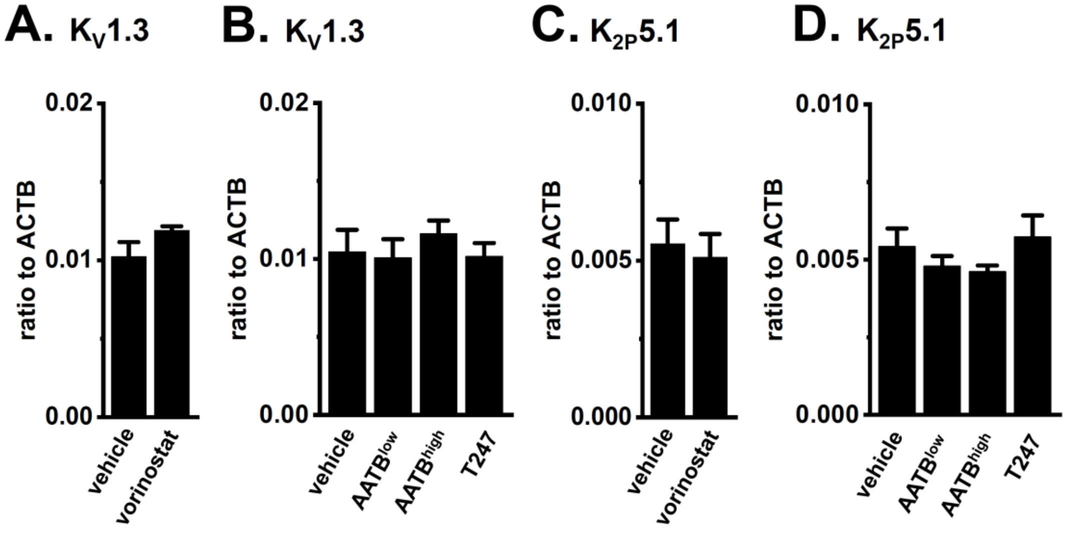

Figure S5. Effects of the treatment with HDACis on the gene expression of $K_V1.3$ and $K_{2P}5.1$ in splenic $CD4^+$ T cells of IBD model mice. A-D: Real-time PCR assay for $K_V1.3$ (**A**,**B**) and $K_{2P}5.1$ (**C**,**D**) in "vehicle"-, 1 μM "vorinostat" (**A**,**C**)-, 30 nM AATB ("AATB^{low}") (**B**,**D**)-, 300 nM AATB ("AATB^{high}") (**B**,**D**)-, and 1 μM "T247" (**B**,**D**)-treated $CD4^+$ T cells (*n* = 4 for each). Expression levels were expressed as a ratio to ACTB. Results are expressed as means ± SEM.

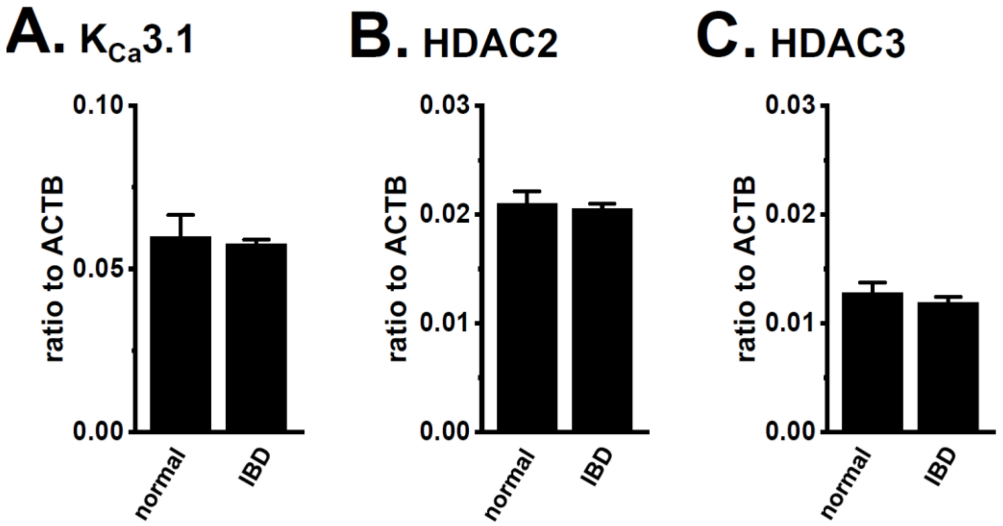

Figure S6. Gene expression of $K_{Ca}3.1$, HDAC2, and HDAC3 in splenic $CD4^+CD25^+$ T cells of normal and IBD model mice. A-C: Real-time PCR assay for $K_{Ca}3.1$ (**A**), HDAC2 (**B**), and HDAC3 (**C**) in the splenic $CD4^+CD25^+$ T cells of "normal" and "IBD" model mice ($n = 4$ for each). Expression levels were expressed as a ratio to ACTB. Results are expressed as means ± SEM.
